# Colon Cancer Cells Gene Expression Signature As Response to 5- Fluorouracil, Oxaliplatin, and Folinic Acid Treatment

**DOI:** 10.3389/fphar.2016.00172

**Published:** 2016-06-23

**Authors:** Carolina Negrei, Ariana Hudita, Octav Ginghina, Bianca Galateanu, Sorina Nicoleta Voicu, Miriana Stan, Marieta Costache, Concettina Fenga, Nikolaos Drakoulis, Aristidis M. Tsatsakis

**Affiliations:** ^1^Department of Toxicology, Faculty of Pharmacy, “Carol Davila” University of Medicine and PharmacyBucharest, Romania; ^2^Department of Biochemistry and Molecular Biology, University of BucharestBucharest, Romania; ^3^Department of Surgery, “Sf. Ioan” Clinical Emergency HospitalBucharest, Romania; ^4^Department II, Faculty of Dental Medicine, “Carol Davila” University of Medicine and PharmacyBucharest, Romania; ^5^Occupational Medicine Section, Department of Biomedical and Dental Sciences and Morphofunctional Imaging, University of MessinaMessina, Italy; ^6^Research Laboratory of Clinical Pharmacology and Pharmacogenomics, School of Health Sciences, Faculty of Pharmacy, National and Kapodistrian University of AthensAthens, Greece; ^7^Department of Toxicology and Forensic Sciences, Medical School, University of CreteHeraklion, Greece

**Keywords:** colorectal cancer, 5-fluorouracil, oxaliplatin, dihydropyrimidine dehydrogenase, gene expression, DPD polymorphism, personalized medicine

## Abstract

5-FU cytotoxicity mechanism has been assigned both to the miss-incorporation of fluoronucleotides into RNA and DNA and to the inhibition of thymidylate synthase. 5-FU is one of the most widely used chemotherapeutic drugs, although it has severe side effects that may vary between patients. Pharmacogenetic studies related to 5-FU have been traditionally focused on the rate-limiting catabolic enzyme, dihydropyrimidine dehydrogenase that breaks 80–85% of 5-FU into its inactive metabolite. Choosing the right dosing scheme and chemotherapy strategy for each individual patient remains challenging for personalized chemotherapy management. In the general effort toward reduction of colorectal cancer mortality, *in vitro* screening studies play a very important role. To accelerate translation research, increasing interest has been focused on using *in vivo-like* models such as three-dimensional spheroids. The development of higher throughput assays to quantify phenotypic changes in spheroids is an active research area. Consequently, in this study we used the microarray technology to reveal the HT-29 colorectal adenocarcinoma cells gene expression signature as response to 5-FU/OXP/FA treatment in a state of the art 3D culture system. We report here an increased reactive oxygen species production under treatment, correlated with a decrease in cell viability and proliferation potential. With respect to the HT-29 cells gene expression under the treatment with 5-FU/OXP/FA, we found 15.247 genes that were significantly differentially expressed (*p* < 0.05) with a fold change higher that two-fold. Among these, 7136 genes were upregulated and 8111 genes were downregulated under experimental conditions as compared to untreated cells. The most relevant and statistic significant (*p* < 0.01) pathways in the experiment are associated with the genes that displayed significant differential expression and are related to intracellular signaling, oxidative stress, apoptosis, and cancer.

## Introduction

Antimetabolite drugs interfere with essential biosynthetic processes or are incorporated into DNA and RNA macromolecules inhibiting their normal function. The fluoropyrimidine 5-fluorouracil (5-FU) does both. Briefly, 5-FU cytotoxicity mechanism has been assigned both to the miss-incorporation of fluoronucleotides into RNA and DNA and to the inhibition of thymidylate synthase (TS). Consequently, 5-FU is converted intracellularly to three active metabolites, namely: fluorodeoxyuridine monophosphate (FdUMP), fluorodeoxyuridine triphosphate (FdUTP), and fluorouridine triphosphate (FUTP) which trigger a series of events that lead to the decrease of cell proliferation potential and induce apoptosis.

Fluoropyrimidines, particularly 5-FU and its pro-drug capecitabine, have been extensively used either as monotherapy or combination therapy for the last five decades worldwide (Ezzeldin and Diasio, 2004) as first line treatment for some solid cancers including gastrointestinal tract and breast (Loganayagam et al., 2010). However, even though the benefits of fluoropyrimidine based chemotherapy are well known, adverse drug reactions are of major clinical concern. Patients receiving 5-FU may experience severe myelosuppression, cardiac toxicity, mucositis, and hand–foot syndrome (Yen and McLeod, 2007) as well as nausea and vomiting. Furthermore, a high level of toxicity (grade 3–4) may encounter in up to 31–34% of patients receiving 5-FU, with 0.5% mortality (Meta-Analysis Group in Cancer et al., 1998).

Capecitabine pro-drug is activated through a multistep process that is transformed to 5-FU preferentially in tumor cells and thus was introduced in breast and colorectal cancer (CRC) chemotherapy to reduce the fluoropyrimidine induced toxicity (van Kuilenburg, 2004). However, 5-FU is one of the most widely used chemotherapeutic drugs, although it has severe side effects that may vary between patients (van Kuilenburg et al., 2003; Lazar et al., 2004; Ciccolini et al., 2006). Choosing the right dosing scheme and chemotherapy strategy for each individual patient remains challenging for personalized chemotherapy management (Ciccolini et al., 2004). To further improve patient outcomes, new regimens containing cytotoxic agents such as oxaliplatin (OXP) and irinotecan have been introduced (de Gramont, 2000). The commonly used combination of these drugs is known as FOLFOX [leucovorin (LV), 5-FU, and OXP] or FOLFILI (LV, 5-FU, and irinotecan).

Though FOLFOX and FOLFIRI treatments have improved survival and response rates in patients with metastatic disease (de Gramont, 2000; Goldberg et al., 2004; Thirion et al., 2004; Van Cutsem et al., 2009), 5-FU-based treatment may be demanding because of patient efficacy and toxicity variability (Diasio and Harris, 1989; McLeod et al., 2003). While variability may be linked to multiple clinical factors, genetic differences might also contribute to drug response.

Pharmacogenetic studies related to 5-FU have been traditionally focused on the rate-limiting catabolic enzyme, dihydropyrimidine dehydrogenase (DPD). DPD is one of the pivotal enzymes in the metabolism pathway of 5-FU, which breaks 80–85% of 5-FU into inactive DHFU metabolite (Diasio and Harris, 1989). Thus, DPD's activity and its gene expression provide a promising instrument to predict the pharmacokinetics and also the chemotherapeutic toxicity of 5-FU.

OXP is an anticancer agent that acts by the formation of Platinum-DNA (Pt-DNA) adducts resulting in DNA-strand breaks (Woynarowski et al., 1998). These Pt-DNA adducts typically are in the form of Pt-Guanine-Guanine (Pt-GG) bonding (Chaney et al., 2004). Ultimately, Pt-DNA complexes at the nucleotide level will either activate DNA repair mechanisms [nucleotide excision repair (NER) pathway] or apoptotic pathways.

Leucovorin or folinic acid (FA, 5′-formyltetrahydrofolate) has been used to expand the intracellular concentration of reduced folate (CH_2_THF) which is necessary for optimal binding of FdUMP, one of the 5-FU's active metabolite, to TS. FA enters the cell via the reduced folate carrier and is anabolized to CH_2_THF, which is then polyglutamated by folylpolyglutamate synthase. Polyglutamation not only increases the cellular retention of CH_2_THF, but also enhances the stabilization of its ternary complex with TS and FdUMP (Dolnick and Cheng, 1978; Radparvar et al., 1989). Furthermore, the Advanced Colorectal Cancer Meta-analysis Project (ACCMP) showed that 5-FU/FA generated significantly superior response rates compared with single agent 5-FU (23 vs. 11%). However, this didn't improve the overall survival (The Meta-Analysis Group in Cancer, 1992).

In the general effort toward reduction of CRC mortality, *in vitro* screening studies play a very important role. Yet, preclinical cancer models predicting clinical treatment outcome are needed in the development of new therapeutic approaches. Nowadays, much effort is being spent on the design of advanced preclinical models that could provide a robust solution to bridge the gap between good preclinical results and success in clinical practice. Standard two-dimensional (2D) cell cultures for effect testing of anticancer agents are simple and convenient, but present significant limitations in reproducing the complexity and pathophysiology of *in vivo* tumor tissue (Galateanu et al., 2016). To accelerate translation research, increasing interest has been focused on using three-dimensional (3D) spheroids for modeling cancer and tissue biology.

Development of higher throughput assays to quantify phenotypic changes in spheroids is an active research area (Galateanu et al., 2016). Furthermore, microarray technology has the potential both to identify novel genes that have key roles in mediating resistance to 5-FU-based chemotherapy and also reveal the gene expression signature of CRC cells as response to 5-FU-based chemotherapy. Such genes might be therapeutically valuable as predictive biomarkers of 5-FU chemosensitivity and/or provide new molecular targets that overcome drug resistance.

In this context, we used the microarray technology to reveal the HT-29 colorectal adenocarcinoma cells gene expression signature as response to 5-FU/OXP/FA treatment in a state of the art 3D culture system.

## Materials and methods

### Cell culture model and drugs treatments

HT-29 human colon adenocarcinoma cells (American Type Culture Collection) were cultured routinely at 37°C under a humidified atmosphere of 5% CO_2_ as a monolayer in Dulbecco's modified Eagle's Medium (DMEM), supplemented with 10% fetal bovine serum (FBS) and 1% penicillin—streptomycin. All the studies were performed using a scaffold free 3D culture system. In this view, multi cellular tumor spheroids (MCTSs) were obtained as previously described (Galateanu et al., 2016) during 4 days of culture after seeding 5 × 10^3^ cells / 20 μl in 384 Perfecta hanging drop culture plates.

For the described experiments the treatments concentrations were previously optimized on this particular 3D culture model (Galateanu et al., 2016). Briefly, on the 5th day of culture some MCTSs were left untreated and some MCTSs were treated with 5-fluorouracil (5-FU, SIGMA Aldrich, code 1001963413), oxaliplatin (OXP, SIGMA Aldrich, code 1001946478) and folinic acid (FA, SIGMA Aldrich, code 101563489) for 24 h, 3 and 7 days, as described in Table 1.

**Table 1:** **MCTSs treatment**.

**Sample**	**Treatment**	**Time**	**Abbreviation**
Control	Untreated	NA	C_MCTSs_
5-FU+OXP+AF	15 mM 5-FU + 500 μM + 2 mM FA	24 h	T_1_MCTSs
5-FU+OXP+AF	15 mM 5-FU + 500 μM + 2 mM FA	3 days	T_2_MCTSs
5-FU+OXP+AF	15 mM 5-FU + 500 μM + 2 mM FA	7 days	T_3_MCTSs

### Live/dead fluorescence microscopy assay

HT-29 MCTSs morphology and dimensions as well as treatments anti-proliferative potential were investigated by Live/Dead (Invitrogen, Foster, CA) fluorescence assay. In this view, C_MCTSs_, T_1_MCTSs, T_2_MCTSs, and T_3_MCTSs were stained for 20 min at room temperature and darkness with a solution containing calceinAM and ethidium bromide, prepared according to the manufacturer's recommendation.

### The measurement of intracellular reactive oxygen species (ROS)

ROS production was assessed using fluorescent 2′,7′-dichlorofluorescein diacetate (DCFH-DA) (Sigma-Aldrich). In this view, C_MCTSs_ and T_3_MCTSs were dissociated by tripsin/EDTA treatment for 15 min at 37°C and the resulting cells were incubated with 10 μM DCFH-DA for 30 min at 37°C. The fluorescence of the resulting dichlorofluoroscein (DCF) was quantified using a fluorimeter (Jasco FP 750), with excitation and emission wavelengths of 485 and 530 nm, respectively.

### Analysis of gene expression by microarray

C_MCTSs_ and T_3_MCTSs were dissociated in individual cells by trypsin/EDTA treatment for 15 min at 37°C and then subjected to RNA isolation and purification using the PureLink RNA Mini Kit (Ambion, Life Technologies, Foster City, CA, USA—Thermo Fisher Scientific), according to the manufacturer's protocols. Further, the RNA samples were tested for integrity on BioAnalyzer 2100 (Agilent Technologies, Waldbronn, Germany) and purity on NanoDrop 8000 spectrophotometer (Thermo Fisher Scientific). Total RNA was converted intro cyanine—3 (Cy3) labeled cRNA using the One—Color Low Input Quick Amp Labelling Kit (Agilent Technologies) according to the manufacturer's instruction, followed by RNAeasy Mini Kit (Qiagen) purification. The cRNA concentration and the dye incorporation were assessed with a NanoDrop 8000 Spectrophotometer (Thermo Fisher Scientific). The samples were hybridized using Agilent Gene Expression Hybridization Kit (Agilent Technologies), following the manufacturer's instruction. Briefly, 600 ng of Cy3—labeled fragmented cRNA was hybridized overnight (65°C, 17 h) to SurePrint G3 Human GE 8 × 60 k microarray slides in a rotating Agilent hybridization oven. After hybridization, microarray slides were washed in Washing Solutions I and II according to the producer's wash buffer kit instructions (Agilent Technlogies) and scanned immediately on the Agilent DNA Microarray Scanner Model G2505C using default one color scan settings. The quantification of the scanned microarray images was done with Feature Extraction Software 12.0 (Agilent Technologies) using default settings.

The obtained data were further exported in GeneSpring GX 13.0 (Agilent Technologies). The raw signals were log transformed and normalized using Quantile normalization method, with a threshold set at 1.0. For each probe, the median of the log summarized value from the control samples was calculated and subtracted from each of the samples to get transformed baseline. The parameter values for experimental grouping were set as treated with 5-FU + OXP + FA and untreated (control probes). Probes that present compromised and non-detected flags were filtered out using the Filter Probesets by flags options available in GeneSpring GX 13.0. The obtained results were further filtered on confidence using Moderated *t*-test with a *p*-value cut-off set at 0.05, using Benjamini—Hochberg multiple testing correction. The results obtained were further used to generate the list of statistically significant differentially regulated genes between treated MCTS and control MCTS, using a fold change higher than two-fold. The graphical representation of the distribution of the fold changes and their associated *p*-values as a volcano plot, was also generated using GeneSpring GX 13.0 (Agilent Technologies). To further characterize the biological pathways that are significant enriched based on the list of statistically significant differentially expressed genes, Single Experiment Analysis in GeneSpring GX 13.0 was performed using a *p*-value cut-off set at 0.05. More, in order to determine biologically significant networks and canonical pathways modulated by our treatment regimen, we exported the list of significant differentially expressed genes (*p* < 0.05) with a fold change higher than 6 from GeneSpring GX 13.0 to Ingenuity Pathway Analysis Software (Qiagen).

### Statistical analysis

The fluorimetric data obtained were statistically analyzed using GraphPad Prism 3.03 Software, one-way ANOVA, Bonferroni test. All the experiments were performed with *n* = 3 biological replicates and each data set is presented as the average of three replicates (mean ± standard deviation). Level of significance was *p* < 0.05.

## Results

### The measurement of intracellular reactive oxygen species (ROS)

**Figure 1:**
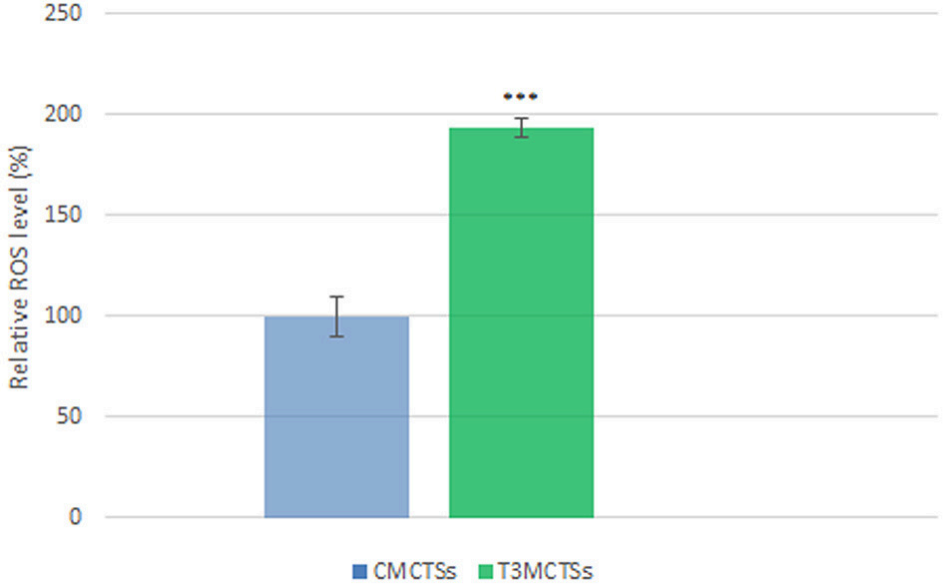
**ROS relative levels in untreated MCTSs (C_**MCTSs**_) and in MCTSs treated for 7 days with 5-FU + OXP + FA (T_**3**_MCTSs)**. ^***^p < 0.001 ROS relative level in T_3_MCTSs vs. C_MCTSs._

Intracellular reactive oxygen species (ROS) production by the HT-29 cells in MCTSs as response to 5-FU + OXP + FA treatment was evaluated in our experimental conditions. Our results show that 5-FU + OXP + FA treatment generated high levels of ROS in HT-29 adenocarcinoma cells, as the T_3_MCTSs displayed a significantly increased (*p* < 0.001) level of ROS as compared with C_MCTSs_ (Figure 1).

This difference in ROS production indicates that the 5-FU + OXP + FA treatment induced the oxidative response which enhanced the formation of a considerable amount of free radicals. The ROS generated could be responsible for damaging effects on proteins and lipids, and could also alter the cell membrane integrity of HT-29 cells, inducing a decrease in cell viability.

**Figure 2:**
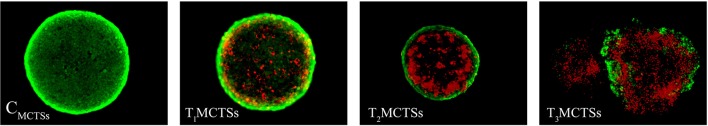
**Fluorescence microscopy images revealing the HT-29 MCTSs stained with calceinAM (green) for living cells and ethidium bromide (red) for dead cells after no treatment (CMCTSs) and after 24 h, 3 and 7 days of exposure to 15 mM 5-FU + 500 μM + 2 mM FA (T1MCTSs, T2MCTSs, and T3MCTSs, respectively)**.

Regarding this particular aspect, we assessed the proliferative status of HT-29 cells in MCTSs as response to 5-FU + OXP + FA treatment by using the Live/Dead fluorescence microscopy assay. In this view, CMCTSs, T_1_MCTSs, T_2_MCTSs, and T_3_MCTSs were double stained with calceinAM and ethidium bromide to highlight both the living and the dead cells in the 3D Spheroids. The images were captured using an Olympus IX73 fluorescence inverted microscope and the CellF software (Figure 2).

The captured images revealed the overall morphology of the MCTSs during 7 days of treatment as well as the distribution of the living and the dead cells inside the 3D structures. Consequently, a compact spheroid consisting in bright green living cells was found in the untreated sample. After 24 h of treatment some dead cells were identified in the core of the MCTSs. Two days later, the diameter of the MCTSs decreased as compared to CMCTSs or T_1_MCTSs and only a thin layer at the periphery displayed live cells. Moreover, after 1 week of treatment we found that the spheroids were disintegrated and the ratio between the living and dead cells was significantly in the favor of the dead cells (Figure 2).

### Microarray gene expression profiling of MCTSs treated with 5-FU + OXP + FA

**Figure 3:**
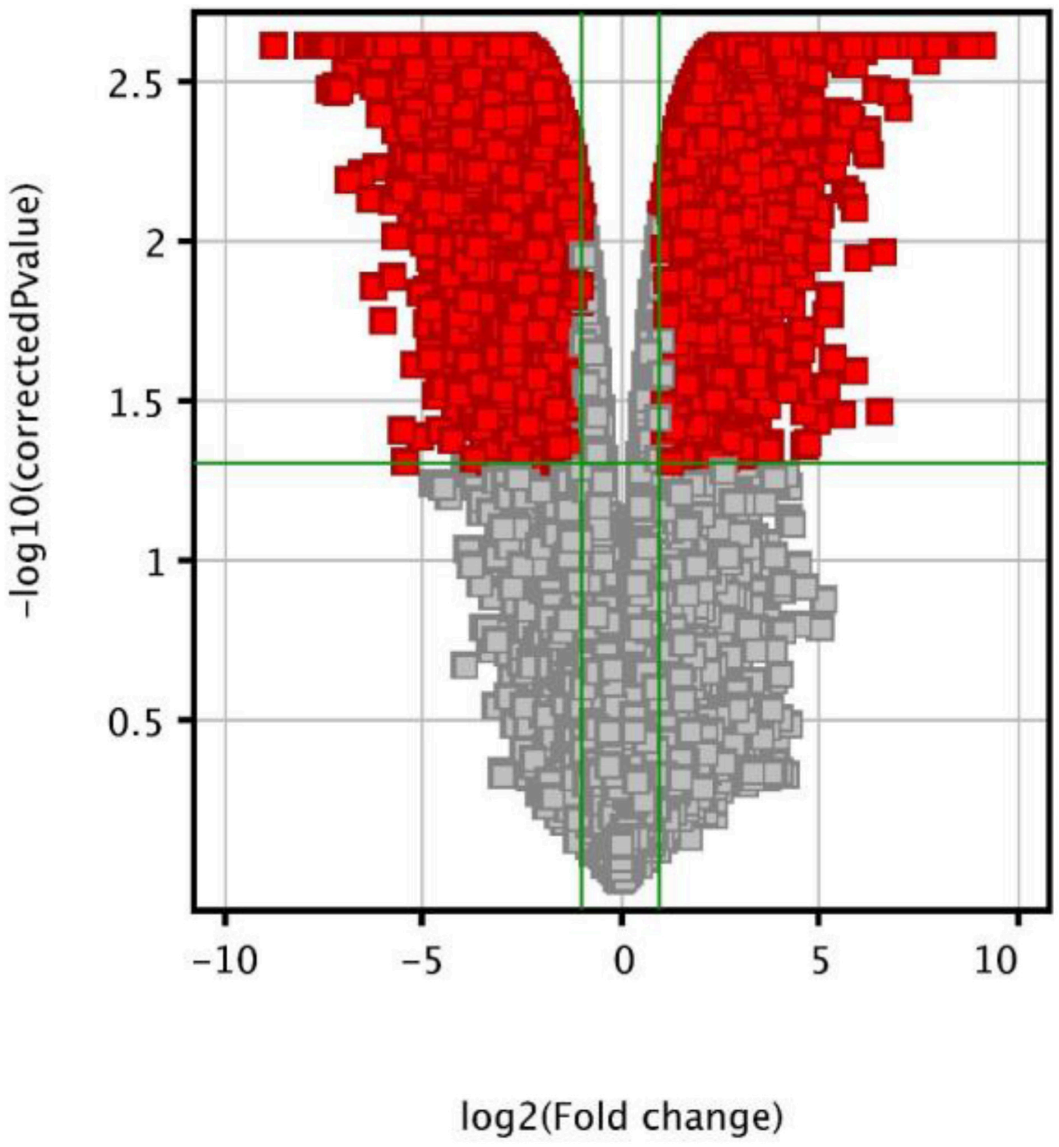
**Volcano plot showing the distribution of the gene expression fold changes of the T_**3**_MCTSs compared with C_**MCTSs**_**. Genes with absolute fold change ≥2 and *p* < 0.05 are indicated in red.

Gene expression microarray analysis was employed to identify the molecular changes occurring during 7 days of MCTSs exposure to 5-FU + OXP + FA treatment (T_3_MCTSs) in comparison with untreated MCTSs (C_MCTSs_). After performing moderated *t*-test analysis with Bonferroni correction, the screening process led to the identification of 15.247 genes that were significantly differentially expressed (*p* < 0.05) with a fold change higher that two-fold. Among these, 7136 genes were upregulated and 8111 genes were downregulated T_3_MCTSs as compared with C_MCTSs_. The distribution of the fold changes and their associated *p*-values in these genes was graphically represented using the volcano plot shown in Figure 3.

Furthermore, we analyzed the effects of 5-FU + OXP + FA treatment on colorectal adenocarcinoma-related key genes in our HT-29 MCTSs study model, focusing on genes correlated with chemosensitivity or prognosis in colorectal adenocarcinoma. Our results show that the expression of critical genes encoding DNA excision repair proteins was significantly decreased by 5-FU + OXP + FA treatment. Such examples include the Excision Repair Cross-Complementation Group 1 (ERCC-1, −4.6-fold) and the Excision Repair Cross-Complementation Group 4 (ERCC-4, −10.6-fold).

5-FU + OXP + FA treatment also influenced the mRNA levels of genes involved in 5-FU metabolic pathway, including dihydropyrimidine dehydrogenase (DPYD, −6.7-fold), ATP-binding cassette sub-family G member 2 (ABCG2, −9.9-fold), carboxylesterase 1 (CES1, +3-fold), uridine phosphorylase 1 (UPP1, +4.1-fold), cytidine deaminase (CDA, −15.7-fold), uridine-cytidine kinase 1 (UCK1, −8.6-fold), uridine monophosphate synthase (UMPS, −2.4-fold), phosphoribosyl pyrophosphate amidotransferase (PPAT, +2.3-fold), thymidine kinase 1 (TK1, −15.5-fold), ATP binding cassette subfamily C member 5 (ABCC5, −15.36-fold), ATP binding cassette subfamily C member 3 (ABCC3, −15.29-fold), methylenetetrahydrofolate reductase (MTHFR, −3.4-fold), folylpolyglutamate synthase (FPGS, −4.1-fold), gamma-glutamyl hydrolase (GGH, −7.8-fold), dihydrofolate reductase (DHFR, −15.1-fold), excision repair cross-complementation group 2 (ERCC2, −11.4-fold), thymine DNA glycosylase (TDG, +4.34-fold), single-strand selective monofunctional uracil DNA glycosylase (SMUG1, −3.4-fold), and thymidylate synthese (TS, −6.7-fold).

### Identification of biological pathways affected by 5-FU + OXP + FA

The most important pathways associated with the genes that displayed significant differential expression between T_3_MCTSs and untreated C_MCTSs_ (*p* < 0.05, fold change ≤2) were identified using the Single Experiment Analysis Tool in GeneSpring GX 13.0 and public pathway database. Consequently, as shown in Table 2, our data reveal that most of the relevant and statistic significant (*p* < 0.01) pathways in the experiment are related to intracellular signaling, oxidative stress, apoptosis, and cancer.

**Table 2:** **Relevant pathways modulated by 5-FU + OXP + FA treatment (***p*** < 0.01)**.

**Pathway name**	***p*-value**	**Matched entities**	**Total pathway entities**
Hs_AMPK_Signaling_WP1403_79471	8.28E-11	45	68
Hs_Apoptosis_Modulation_and_Signaling_WP1772_80459	2.42E-08	52	95
Hs_Apoptosis_WP254_80450	4.17E-09	50	87
Hs_Benzo(a)pyrene_metabolism_WP696_72081	0.0090	6	9
Hs_DNA_Damage_Response_WP710_79974	8.78E-09	62	114
Hs_DNA_Damage_Reversal_WP1804_83251	0.0089	5	6
Hs_EGF-EGFR_Signaling_Pathway_WP437_79266	3.82E-09	82	162
Hs_Fluoropyrimidine_Activity_WP1601_82222	2.16E-07	24	34
Hs_Folate-Alcohol_and_Cancer_Pathway_WP1589_82223	0.00901	6	8
Hs_Gastric_Cancer_Network_1_WP2361_84551	3.62E-06	20	29
Hs_Integrated_Cancer_Pathway_WP1971_82939	4.51E-05	26	49
Hs_MAPK_Signaling_Pathway_WP382_79951	3.84E-11	89	168
Hs_Oncogene_Induced_Senescence_WP3308_83246	0.0098	12	26
Hs_Oxidative_Stress_Induced_Senescence_WP3404_83222	7.05E-07	49	99
Hs_Oxidative_Stress_WP408_78546	3.54E-04	18	30
Hs_Senescence_and_Autophagy_in_Cancer_WP615_81193	5.87E-04	47	109

*The matched entity value represents the total number of genes differentially expressed in a pathway in given experimental conditions compared with the total number of entities of the pathway in the database*.

In order to assess the molecular functions and canonical pathways modulated by 5-FU + OXP + FA treatment, the significant differentially expressed genes (*p* < 0.05) with a fold change >6 were uploaded to Ingenuity Pathway Analysis (IPA). The analysis of the canonical pathways revealed that a large number of canonical pathways are modulated by the treatment with 5-FU + OXP + FA. The top five pathways are represented in Table 3, along with their respective *p*-values and ratio of the number of genes in the differential expression gene list under given experimental conditions over the total number of genes found in the respective canonical pathway.

**Table 3:** **Top five canonical pathways identified with Ingenuity Pathway Analysis (IPA) in T_**3**_MCTSs**.

**Canonical Pathway**	***P*-value**	**Ratio**
Molecular mechanism of cancer	2.44E−07	0.236
Xenobiotic metabolism signaling	6.9E−05	0.223
Noradrenaline and adrenaline degradation	7.68E−05	0.395
Glycogen degradation II	1.82E−04	0.636
Putrescine degradation III	1.95E−04	0.476

*The ratio value refers to the number of genes in the differential expression gene list under given experimental conditions over the total number of genes found in the respective canonical pathway*.

Furthermore, IPA analysis showed that the most significant cellular and molecular functions affected by 5-FU + OXP + FA treatment in HT-29 cells grown in MCTSs were related to cancer, organismal injury and abnormalities, gastrointestinal diseases, cell cycle and cellular growth, and proliferation.

**Figure 4:**
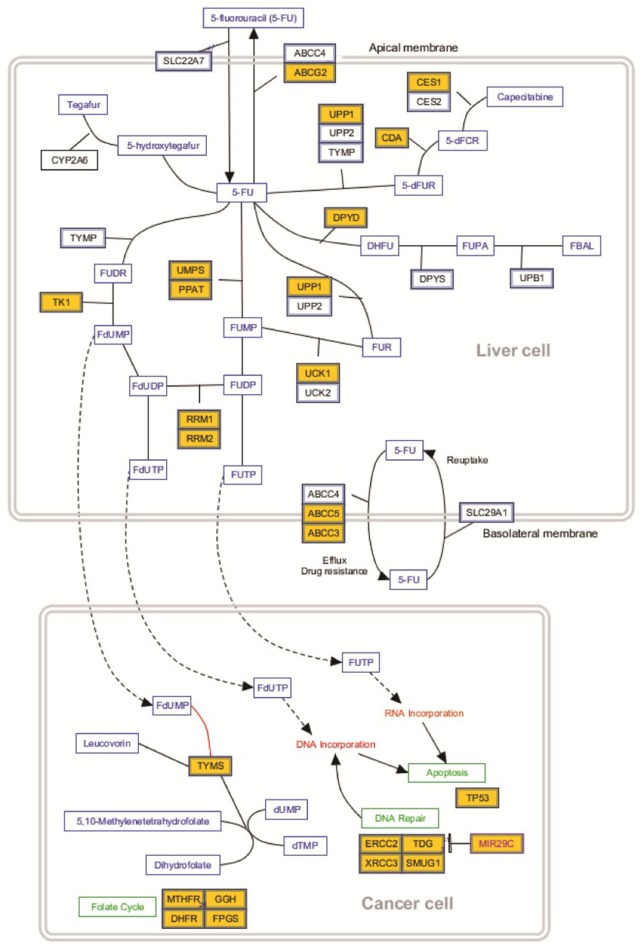
**5-FU metabolism**. Genes significantly up- or down-regulated by 5-FU + OXP + FA are represented in yellow boxes: ABCC2, ATP binding cassette subfamily C member 2; CES1, carboxylesterase 1; UPP1, uridine phosphorylase 1; CDA, cytidine deaminase; DPD, dihydropyrimidine dehydrogenase; UMPS, uridine monophosphate synthase; PPAT, phosphoribosyl pyrophosphate amidotransferase; TK1, thymidine kinase 1; UCK1, uridine-cytidine kinase; ABCC5, ATP binding cassette subfamily C member 5; ABCC3, ATP binding cassette subfamily C member 3; TYMS, thymidylate synthase; ERCC2, excision repair cross—complementation group 2; TDG, thymine DNA glycosylase; SMUG1, single-strand selective monofunctional uracil DNA glycosylase; MTHFR, methylenetetrahydrofolate reductase; GGH, gamma—glutamyl hydrolase; DHFR, dihydrofolate reductase; and FPGS, folylpolyglutamate synthase.

In our experiment, after 7 days of treatment with 5-FU + OXP + FA, PPAT gene expression in T_3_MCTSs was significantly upregulated as compared to C_MCTSs_, while UMPS gene expression was found significantly downregulated (Figure 4). Furthermore, only UPP1 and UCK1 genes expression were found significantly upregulated and downreguated, respectively. TK1 gene expression was found in our experimental conditions highly downregulated (Figure 4). Additionally, we report a decrease in DPYD gene expression in T_3_MCTSs as compared to C_MCTSs_ (Figure 4). Nevertheless, ERCC1 gene expression was significantly downregulated in T_3_MCTSs as compared to C_MCTSs_.

Consequently, at a global level, the genes involved in this processes are down regulated in T_3_MCTSs as compared with the C_MCTSs_.

## Discussions

5-FU is converted in the hepatocytes into three main active metabolites: fluorodeoxyuridine monophosphate (FdUMP), fluorodeoxyuridine triphosphate (FdUTP), and fluorouridine triphosphate (FUTP). The main mechanism of 5-FU activation is conversion to fluorouridine monophosphate (FUMP), either directly by phosphoribosyl pyrophosphate amidotransferase (PPAT) and uridine monophosphate synthase (UMPS) or via fluorouridine (FUR) through the sequential action of uridine phosphorylase (UPP) and uridine kinase (UCK). An alternative activation pathway involves the thymidine phosphorylase catalyzed conversion of 5-FU to fluorodeoxyuridine (FUDR), which is then phosphorylated by thymidine kinase (TK) to FdUMP. Next, FUMP is phosphorylated to fluorouridine diphosphate (FUDP), which can be either further phosphorylated to the active metabolite fluorouridine triphosphate (FUTP), or converted to fluorodeoxyuridine diphosphate (FdUDP) by ribonucleotide reductase (RRM). In turn, FdUDP can either be phosphorylated or dephosphorylated to generate the active metabolites FdUTP and FdUMP, respectively.

Dihydropyrimidine dehydrogenase (DPD)-mediated conversion of 5-FU to dihydrofluorouracil (DHFU) (Heggie et al., 1987) is the rate-limiting step of 5-FU catabolism in normal and tumor cells. Up to 85% of administered 5-FU is catabolized by DPD in the liver. However, genetic polymorphisms in DPYD results in wide inter-individual variation in DPD activity (Etienne et al., 1995). A patient with partial or complete DPD deficiency will accumulate active metabolites and could suffer serious toxicity or even rare fatality, when exposed to fluoropyrimidines. Consequently, the genetic approach to DPD-associated toxicity was considered promising, as deleterious variants were found in the DPD coding gene (*DPYD*, chromosome 1p22). Three DPYD variants (IVS14 + 1G>A, c.2846A>T, and c.1679T>G) were associated with fluoropyrimidine-induced toxicity (van Kuilenburg, 2004). Furthermore, subsequent analysis on large cohorts of patients suffering from 5-FU toxicity and control individuals demonstrated that DPYD has been previously reported as highly polymorphic (van Kuilenburg, 2004). *In vitro* studies revealed that DPD overexpression in cancer cell lines is correlated with 5-FU resistance (Takebe et al., 2001). Furthermore, high levels of DPD mRNA expression in CRC have also been demonstrated to interact with resistance to 5-FU (Salonga et al., 2000), probably due to the higher DPD-mediated degradation of 5-FU in these tumors. Additionally, DPD, TS, and Thymidine phosphorylase (TP) might be considered independent predictive markers of 5-FU response and that the measurement of all these three markers markedly enhanced the possibility to predict tumor response to 5-FU-based chemotherapy (Salonga et al., 2000).

The reductive methylation of deoxyuridine monophosphate (dUMP) to deoxythymidine monophosphate (dTMP) is catalyzed by TS, while the reduced folate 5,10-methylenetetrahydrofolate (CH_2_THF) is acting as the methyl donor (Figure 4). This reaction provides the only *de novo* source of thymidylate, which is necessary for DNA replication and repair. The 36-kDa TS protein functions as a dimer, with two subunits containing a nucleotide-binding site and a binding site for CH_2_THF. The 5-FU metabolite FdUMP binds to the nucleotide-binding site of TS, forming a stable ternary complex with the enzyme and CH_2_THF, thereby blocking binding of the normal substrate dUMP and inhibiting dTMP synthesis (Santi et al., 1974; Sommer and Santi, 1974).

Preclinical studies have demonstrated that TS expression is a key determinant of 5-FU sensitivity. Gene amplification of TS with consequent increase in TS mRNA and protein has been observed in cell lines that are resistant to 5-FU and fluorodeoxyuridine (FUDR) (Johnston et al., 1992; Copur et al., 1995). In our study we found TS significantly downregulated in T_3_MCTSs as compared to C_MCTSs_.

The 5-FU metabolite FUTP is extensively incorporated into RNA, disrupting normal RNA processing and function. Significant correlations between 5-FU miss-incorporation into RNA and loss of clonogenic potential have been shown in human colon and breast cancer cell lines (Kufe and Major, 1981; Glazer and Lloyd, 1982). 5-FU miss-incorporation can result in toxicity to RNA at several levels. It not only inhibits the processing of pre- rRNA into mature rRNA (Kanamaru et al., 1986; Ghoshal and Jacob, 1994), but also disrupts post-transcriptional modification of tRNAs (Randerath et al., 1983; Santi and Hardy, 1987) and the assembly and activity of snRNA/protein complexes, thereby inhibiting splicing of pre-mRNA (Doong and Dolnick, 1988; Patton, 1993). In addition, rRNA, tRNA, and snRNA all contain the modified base pseudouridine, and 5-FU has been shown to inhibit the post-transcriptional conversion of uridine to pseudouridine in these RNA species (Samuelsson, 1991). Consequently, 5-FU miss-incorporation can potentially disrupt many aspects of RNA processing, leading to profound effects on cellular metabolism and viability. At this level, our results show a significant decrease of ERCC2 and SMUG1 genes expression, with high impact on cell viability and proliferation status.

The exact mechanism of synergism between 5-FU and OXP is complex, but experimental observations suggest that OXP can downregulate or inhibit DPD, slowing the catabolism of 5-FU (Fischel et al., 2002). Despite initial sensitivity to OXP, most cancer cells will eventually develop resistance. The most important mechanisms in OXP resistance seem to be related to DNA repair: MMR, or nucleotide excision repair (NER). NER pathway include excision repair cross-complementing group 1 protein (ERCC1), xerodermapigmentosum complementation group D protein (XPD, also known as ERCC2), glutathione S-transferase P1 (GSTP1), and TS (Martin et al., 2008). Cells that overexpress ERCC1, are resistant to OXP.

Some recent studies (Oka et al., 2012) report on the 5-FU induced gene mutation and chromosomal damage in TP53 mutated cells, but not in TP53 wild-type cells after 24 h of treatment, probably due to the difference in gene expression related to TP53 pathway, especially the induction of apoptosis or cell cycle arrest after DNA damage. Additionally, according to Gherman et al. (2012), the p53 expression in the OXP treated cells results in antitumor effects that include inhibition of cell cycle progression and induction of apoptosis through the modulation of the expression of apoptosis and cell-cycle-related genes, and the sensitization of tumor cells to chemotherapy.

## Conclusions

In the present study, we demonstrated that 5-FU/OXP/FA treatment inhibits HT-29 CRC cells growth within a modern *in vivo-like* 3D culture system, in a time dependent manner, sensitizing these cells to the chemotherapeutic agent in the case of multiple dose administration strategy. This lead to a significant decrease of the viable cells number and also in the microtumor's diameter reduction. The underlying mechanisms of the treatment effects mainly involve the suppression of the proliferative status and the induction of the apoptosis.

With respect to the HT-29 cells gene expression under the treatment with 5-FU/OXP/FA, we found 15,247 genes that were significantly differentially expressed (*p* < 0.05) with a fold change higher that two-fold. Among these, 7136 genes were upregulated and 8111 genes were downregulated under experimental conditions as compared to untreated cells.

The most relevant and statistical significant (*p* < 0.01) pathways in the experiment were found to be associated with intracellular signaling, oxidative stress, apoptosis, and cancer related genes that displayed differential expression.

## Author contributions

CN and OG designed the study and partially edited the manuscript. BG assessed the antiproliferative status of the cells and contributed to the microarray experiment. She also had a substantial contribution in the manuscript drafting. AH performed the microarray experiment and data analysis. SV assessed the ROS production in tumor cells. MC, MS, CF, and ND were involved in manuscript editing. AT was responsible for the coordination of the study and assured a good collaboration between the authors.

### Conflict of interest statement

The authors declare that the research was conducted in the absence of any commercial or financial relationships that could be construed as a potential conflict of interest.

## Abbreviations

2D: two-dimensional
3D: three-dimensional
5-FU: 5-fluorouracil
ABCC3: ATP binding cassette subfamily C member 3
ABCC5: ATP binding cassette subfamily C member 5
ABCG2: ATP-binding cassette sub-family G member 2
ACCMP: advanced colorectal cancer meta-analysis project
CDA: cytidine deaminase
CES1: carboxylesterase 1
CH2THF: folate
CRC: colorectal cancer
Cy3: cyanine-3
DCFH-DA: 2′, 7′-dichlorofluorescein diacetate
DHFR: dihydrofolate reductase
DHFU: dihydrofluorouracil
DMEM: Dulbecco's modified Eagle‘s Medium
DNA: Deoxyribonucleic acid
DPD: dihydropyrimidine dehydrogenase
DPYD: dihydropyrimidine dehydrogenase
ERCC1: Excision Repair Cross-Complementation Group 1
ERCC2: excision repair cross-complementation group 2
ERCC4: Excision Repair Cross-Complementation Group 4
FA: folinic acid (5′-formyltetrahydrofolate)
FBS: fetal bovine serum
FdUMP: fluorodeoxyuridine monophosphate
FdUTP: fluorodeoxyuridine triphosphate
FPGS: folylpolyglutamate synthase
FUDP: fluorouridine diphosphate
FUMP: fluorouridine monophosphate
FUR: fluorouridine
FUTP: fluorouridine triphosphate
GGH: gamma-glutamyl hydrolase
GSTP1: glutathione S-transferase
IPA: Ingenuity Pathway Analysis
LV: leucovorin
MCTSs: multi cellular tumor spheroids
MTHFR: methylenetetrahydrofolate reductase
NER: nucleotide excision repair
OXP: oxaliplatin
PPAT: phosphoribosyl pyrophosphate amidotransferase
Pt-DNA: Platinum-DNA
Pt-GG: Pt-Guanine-Guanine
RNA: Ribonucleic acid
ROS: reactive oxygen species
SMUG1: single-strand selective monofunctional uracil DNA glycosylase
TDG: thymine DNA glycosylase
TK1: thymidine kinase 1
TS: thymidylate synthase
UCK1: uridine-cytidine kinase 1
UMPS: uridine monophosphate synthase
UPP1: uridine phosphorylase 1.
